# Metabolites and Whole-Genome Analysis of the Lichenysin-Producing *Bacillus licheniformis* YC7

**DOI:** 10.3390/foods15142548

**Published:** 2026-07-19

**Authors:** Xun Liu, Zhen Tang, Han Li, Wenjing He, Yu Chen, Baoyi Guan, Wenli Quan

**Affiliations:** 1 School of Food and Liquor Engineering, Sichuan University of Science & Engineering, Yibin 644000, China; xunliu0123@hotmail.com (X.L.);; 2 Brewing Science and Technology Key Laboratory of Sichuan Province, Sichuan University of Science & Engineering, Yibin 644000, China; 3 Liquor Making Biotechnology and Intelligent Manufacturing of Key Laboratory of China National Light Industry, Yibin 644000, China; 4 Sichuan Province Yibin Changxing Liquor Industry Group Co., Ltd., Yibin 644105, China

**Keywords:** lichenysin, *Bacillus licheniformis*, whole-genome sequencing, metabolites, gene annotation

## Abstract

Lichenysin is a high-performance, environmentally friendly biosurfactant that also has antibacterial activity and flavor-regulating functions. During the solid-state fermentation of Baijiu, lichenysin plays key roles in inhibiting unwanted microbes and stabilizing quality. This study aims to explore the metabolic mechanisms and application potential of lichenysin-producing bacteria. High surfactant-producing strain *Bacillus licheniformis* YC7 was selected through the oil spreading assay, and tandem mass spectrometry (MS/MS) confirmed that its fermentation broth contains multiple lichenysin homologs. Gas chromatography–mass spectrometry (GC-MS) analysis showed that strain YC7 can produce 35 volatile flavor compounds, with pyrazine compounds being the main components. Whole-genome sequencing revealed that the genome size of YC7 was 4.31 Mb, containing 4590 protein-coding genes. A total of 10 secondary metabolite synthesis gene clusters were predicted, among which the complete lichenysin synthesis gene cluster was included. Gene annotation and functional analysis identified key genes and metabolic pathways related to the synthesis of pyrazine, acetoin, and 2,3-butanediol, indicating a high correlation between the genome and metabolome of strain YC7. In summary, this study revealed that strain YC7 had the dual potential to produce both biosurfactants and flavor compounds, providing a theoretical basis for its targeted applications.

## 1. Introduction

*Bacillus licheniformis* is a type of Gram-positive, spore-forming facultative anaerobic bacterium, widely distributed in soil, fermentation systems, and the micro-ecological environments of animals and plants. *B. licheniformis* has strong stress resistance and favorable biosafety and has been generally recognized as safe by the U.S. Food and Drug Administration (FDA) [1]. This type of *Bacillus* can synthesize various functional metabolites during the fermentation process, such as antimicrobial substances (bacteriocins [2], lipopeptides [3,4], and antibiotics [5]), industrial enzymes (amylases [6], cellulases [7], and proteases [8]), and biopolymeric materials (γ-polyglutamic acid [9] and polysaccharides [10,11]), and are widely used in industrial production, food brewing, biomedicine, and environmental management [12,13].

Lichenysin is a type of cyclic lipopeptide biosurfactant synthesized by *B. licheniformis* through the non-ribosomal peptide synthetase (NRPS) pathway. Its structural feature is a heptapeptide ring connected at the N-terminus to a β-hydroxy fatty acid chain, which is highly homologous to surfactin, but it has unique physicochemical properties due to the replacement of glutamic acid with glutamine at the first position of the peptide ring [14,15]. Early studies mainly focused on its surface activity (such as reducing the surface tension of the aqueous phase) and its potential applications in industrial fields such as oil recovery and environmental remediation [14,15]. In recent years, research has found that lichenysin is widely present in the fermentation system of traditional Chinese Baijiu and is a key factor involved in the regulation of Baijiu flavor [16,17]. Zhang et al. [3] found that lichenysin can significantly improve the aromatic quality and coordination of Baijiu by selectively regulating the volatility of aromatic substances. It has been reported that lichenysin can indirectly promote the conversion of flavor precursor substances and the accumulation of characteristic aromas by improving the wettability of microbial surfaces and enhancing microbial colonization ability [18,19]. Lichenysin, as a non-volatile macromolecule, can selectively bind volatile odor substances such as phenol, 4-ethylphenol, and 4-ethylguaiacol through hydrogen bonding and hydrophobic interactions, significantly reducing their headspace concentration (inhibition rate 36~48%) and sensory intensity, while having a minimal effect on the release of major flavor compounds such as esters and alcohols [16]. In addition, the lipopeptide substances, such as lichenysin and surfactin, have a significant antagonistic effect on geosmin-producing *Streptomyces*, which can inhibit the formation of undesirable flavors such as earthy taste, while also ensuring the stability of the fermented microbial community [20,21].

The lichenysin biosynthetic gene cluster and key modules have been reported, and its core synthetic element is the *licA* operon, which can encode the non-ribosomal peptide synthetase (NRPS). The *licA* operon exhibits natural length polymorphism (26.6~32.4 kb) in different *B. licheniformis* strains [14]. The thioesterase (TE) module at the terminus of the *licA* operon is a critical functional node of this pathway, and its core protein LchAD belongs to type II thioesterase (TE II). Studies have found that LchAD not only catalyzes intramolecular cyclization of the lipopeptide chain and release of the final product but also regulates the molecular structure of lichenysin via specific substrate recognition. Additionally, LchAD possesses an editing function that can correct incorrectly activated amino acids, ensuring the catalytic fidelity of the synthetase [22]. In addition, the biosynthesis of lichenysin is also regulated by quorum sensing factors, such as the ComQXPA system [23,24,25]. As the cell density increases during the fermentation process, the ComX pheromone modified by ComQ accumulates extracellularly. Once it reaches a threshold, it binds to the membrane sensor ComP, triggering its autophosphorylation. The phosphate group is then transferred to the response regulator protein ComA, leading to its phosphorylation [23]. Phosphorylated ComA can specifically bind to the promoter region of the *licA* operon, efficiently initiating the transcription of the lichenysin synthesis genes [24,25].

In the solid-state fermentation system of Baijiu, *B. licheniformis* can not only produce non-volatile lipopeptides but also synthesize volatile substances such as pyrazines, ethyl esters, 2,3-butanedione, and volatile organic acids. Among them, pyrazines are the core components of the typical aromas of sauce-flavored and medicinal-flavored Baijiu [17,18]. The metabolic pathways of flavor compounds (especially pyrazines and acetoin) produced by *B. licheniformis* have been reported [17,26]. In addition, existing studies have analyzed the metabolic potential of *B. subtilis* using whole-genome sequencing. It has been reported that a *B. subtilis* strain was isolated from Moutai *Daqu*, and its flavor compound biosynthesis pathways and related genes were predicted through whole-genome sequencing analysis [27]. Researchers isolated a thermophilic *B. subtilis* strain that produced high levels of protease [28]. After conducting a whole-genome sequencing analysis, it was found that this strain also had the potential to synthesize fengicin, lichenysin and surfactin [28]. However, existing studies are mostly limited to the characterization of a single product (lipopeptide or flavor compound), lacking systematic research on the systematic correlation between lichenysin and flavor metabolism within the same strain. Especially at the level of genome and metabolome coupling, comprehensive research on lichenysin-producing *B. licheniformis* to reveal its volatile flavor profile, genomic characteristics, and synthetic pathways is still relatively limited.

Based on the above issues, this study will focus on a lichenysin-producing strain *Bacillus licheniformis* YC7 isolated from *Daqu*, aiming to identify the types of lichenysin produced by this strain through tandem mass spectrometry analysis, to detect and analyze the composition characteristics of its volatile metabolites using GC-MS, and finally to systematically analyze its metabolic product synthesis potential in combination with whole-genome sequencing technology.

## 2. Materials and Methods

### 2.1. Isolation and Culture of Bacillus

Two independent batches of Daqu were collected, and samples from each batch were taken according to different locations in the storage heap. The collected Daqu was crushed and mixed evenly for subsequent research. A total of 5 g of mixed *Daqu* sample was weighed, 45 mL of sterilized saline was added, it was enriched at 37 °C and 180 r/min for 30 min, and then treated in a water bath at 85 °C for 15 min [22]. Then, 10 mL of the water-bath-treated liquid was added to 100 mL of LB liquid medium and cultured at 37 °C and 180 r/min for 24 h to obtain the enriched solution. Taking 20 mL of *Huangshui* (by-products of Baijiu brewing), it was centrifuged at 5000 rpm for 10 min. Taking 10 mL supernatant, it was diluted with sterile water to 50 mL, then incubated in an 85 °C water bath for 15 min [22]. Then, 10 mL of the liquid was taken and inoculated into 100 mL LB liquid medium (10 g/L peptone, 5 g/L yeast extract, and 10 g/L NaCl, with a pH of 7.0, and sterilized at 121 °C for 20 min), and cultured at 37 °C and 180 r/min for 24 h to obtain the enriched solution. Sterile water was used to perform serial dilutions of the enriched solution (dilution of enriched solution obtained from *Daqu*: 10^−5^, 10^−6^, 10^−7^; dilution of enriched solution from *Huangshui*: 10^−3^, 10^−4^, 10^−5^), then spread onto LB agar plates and statically incubated at 37 °C for 24 h. The single colonies with different morphologies were piked for streaking, and repeated multiple times until pure strains were obtained. All purified single colonies were taxonomically identified by 16S rDNA sequencing using the universal bacterial primer pairs 27F/1492R. Whole-genome sequencing was additionally conducted on target strain YC7 to reconfirm its species classification. The isolated strains were numbered and stored for later use.

### 2.2. The Oil Spreading Experiment

The ability of the isolated strains to produce biosurfactants in liquid fermentation was detected using the oil spreading method. Firstly, the liquid fermentation broth was prepared as follows: the strain to be tested was inoculated into 100 mL LB liquid medium and cultured at 37 °C and 180 r/min until the optical density at 600 nm (OD_600_) reached 0.8–1.0 to obtain the seed culture. The seed culture was then inoculated into the fermentation medium (20 g glucose, 15 g soybean tryptone, 2 g KH_2_PO_4_, 0.5 g MgSO_4_·7H_2_O, and 0.01 g FeSO_4_·7H_2_O were dissolved in 1 L distilled water, with a pH of 7.0, and sterilized at 121 °C for 20 min) at 3% (*v*/*v*) and cultured at 37 °C and 200 r/min for 72 h. After fermentation, the fermentation broth was centrifuged at 4 °C, 10,000 r/min for 40 min, and the supernatant was collected for later use. Secondly, the oil spreading experiment was performed according to the method reported by Lyu et al. [29]. Specifically, 30 mL deionized water was added to a 9 cm diameter Petri dish; then, 1 mL Oil Red solution was added. The system was kept static at room temperature for 5 min. After the oil film spread evenly, 2.5 μL of the fermentation supernatant of the test strain was added to the center. After letting it sit for 10 min until the oil displacement ring size stabilized, its diameter was measured. All experiments were performed in triplicate with matched positive and negative controls. Strain YC7, confirmed to produce lichenysin via mass spectrometry [22], served as the positive control for biosurfactant production, while a fermented-soybean-derived *Bacillus paralicheniformis* with no surface activity and oil-displacing capacity (isolated and preserved in our lab) was applied as the negative control.

### 2.3. Tandem Mass Spectrometry Detection

The tandem mass spectrometry method was employed to qualitatively identify the types of biosurfactants in the liquid fermentation products of strain YC7. Firstly, the fermentation broth was pre-treated [22]. The fermentation broth was centrifuged at 8000 r/min for 20 min at 4 °C, and the supernatant was collected. The pH of the supernatant was adjusted to 2.0 using 6 mol/L HCl solution, and it was left at 4 °C overnight. The sediment was then centrifuged at 8000 r/min for 20 min and collected, and the sediment was extracted three times with 30 mL methanol, each extraction being carried out at 4 °C for 4 h. The extracted solutions were collected and filtered through a 0.22 μm microporous membrane. The processed samples were used for tandem mass spectrometry analysis, with the detection conditions set as DP 167 V, EP 6.9 V, CE 40 V, and needle pump flow rate 0.07 mL/min. The scanning range was 100–1100 Da for the primary mass spectrum and 100–1000 Da for the secondary mass spectrum [22]. Liquid chromatography–tandem mass spectrometry (LC-MS/MS) analysis was performed on a QTRAP 6500 triple-quadrupole mass spectrometer (AB Sciex, supplied by Shanghai Guiyi Biochemical Technology Co., Ltd., Shanghai, China). The instrument was equipped with a Turbo Spray electrospray ionization source operated in positive ion mode (ESI+), matched with a triple-quadrupole mass analyzer. Reversed-phase chromatographic separation was conducted with a binary mobile phase system: mobile phase A, 0.1% formic acid aqueous solution; mobile phase B, methanol supplemented with 0.1% formic acid. The injection volume was 5 μL, and linear gradient elution was carried out at a constant flow rate of 0.3 mL/min. Compound identification of lichenysin biosurfactants was realized by matching MS/MS fragment spectra against the Metlin lipopeptide database and NCBI PubChem compound spectral library, combined with published reference secondary mass spectra of lichenysin homologues. Peaks with mass error below 5 ppm and fragment matching scores over 80 were positively identified as target lichenysin analogues.

### 2.4. Analysis of Volatile Metabolites by GC-MS

The strain YC7 was inoculated into LB liquid medium and cultured on a shaker at 37 °C and 120 r/min for 1 day to prepare the seed culture. The seed culture was then inoculated into wheat solid fermentation medium at an inoculation ratio of 10% (*v*/*w*, seed liquid/wheat substrate), followed by static incubation for 5 days. The incubation temperature was increased stepwise every 24 h, with sequential temperatures of 35 °C, 40 °C, 45 °C, 50 °C and 55 °C. For the control group, an equal volume of sterile blank LB liquid medium was added without strain inoculation, and all other fermentation conditions remained identical. A total of 15.0 g fermented solid sample was accurately weighed and transferred into a 50 mL headspace vial. The sample was heated in a 60 °C water bath for 15 min, followed by 30 min headspace adsorption prior to GC-MS analysis. The overall detection procedure was performed according to the method reported by Zhang et al. [30], and 2-ethylbutyric acid was selected as the internal standard for semi-quantitative calculation of volatile compounds.

In addition, the detailed instrumental parameters were set according to the method reported by Liu et al. [31]. Briefly, a DB-WAX capillary column (60 m × 250 µm, 0.25 µm) was adopted; the inlet temperature was 230 °C with a programed oven temperature ramp from 40 °C to 230 °C. The MS scan range was 20–500 u at 70 eV EI energy, and the ion source and transfer line were both held at 230 °C. Qualitative identification and semi-quantitative analysis: All volatile flavor compounds were identified by matching their mass spectra against the NIST standard mass spectral library. Compounds with a spectral similarity score higher than 80% were regarded as reliable target substances. Semi-quantitative analysis was conducted based on the peak area ratio of each target compound to the internal standard (2-ethylbutyric acid) to calculate the absolute content of each volatile component.

### 2.5. Whole-Genome Sequencing and Assembly

The strain YC7 cultivated to the logarithmic phase was centrifuged at 10,000 rpm for 10 min at 4 °C to collect the cells. An appropriate amount of 1 × PBS buffer was added to wash the cells three times, followed by centrifugation at 10,000 rpm for 10 min at 4 °C to collect the cells. The cells were rapidly frozen in liquid nitrogen for more than 15 min and transported on dry ice for testing. Whole-genome shotgun (WGS) sequencing was commissioned to Shanghai Personal Biotechnology Co., Ltd. (Shanghai, China). Two libraries were constructed following official kits: a 400 bp paired-end library with Illumina TruSeq Nano DNA LT Kit for Illumina NovaSeq platform and a long-read library with PacBio Template Prep Kit 1.0 for PacBio Revio platform; large fragments were recovered using BluePippin system during PacBio library preparation. Raw Illumina reads were trimmed for adapters by AdapterRemoval and filtered for low-quality reads using SOAPec as recommended by the standard pipeline. Short-read contigs were assembled with SPAdes and A5-miseq. PacBio long reads were separately assembled with Flye v2.9.1 and Unicycler v0.5.0. All assembled contigs from short and long reads were integrated, and Pilon software (v1.24) was used to polish the consensus sequence with high-quality Illumina data to obtain a complete circular bacterial chromosome. The hybrid PacBio and Illumina sequencing achieved an average sequencing depth of 265×.

### 2.6. Gene Annotation and Functional Analysis

Genome circular maps were drawn using CGview online server Version 2020 (https://proksee.ca) (accessed on 23 July 2025). By aligning the genome sequence with the Non-Redundant Protein Database (NR, v2023.02), the species information of the genome was obtained. Protein-coding genes were predicted using the eggnog-mapper software (Version v2.1.12) to obtain COG annotations; based on the GO IDs in the interproscan prediction results, GO annotations were performed using map2slim (https://metacpan.org/dist/go-perl/view/scripts/map2slim) (accessed on 23 July 2025). KO and pathway annotations of protein-coding genes were mainly completed using KEGG’s KAAS automated annotation system, analyzing their metabolic pathways and the relationships between pathways. The hmmscan software (Version v3.4) was used to align sample gene-encoded protein sequences with the CAZy database (http://www.cazy.org/) (accessed on 23 July 2025) to predict CAZy enzyme genes present in the genome sequence. Secondary metabolite gene clusters were predicted using antiSMASH software (Version v7.1.0.). BLAST software (Version v2.13.0) was used to compare gene-encoded protein sequences against the antibiotic resistance gene database (https://card.mcmaster.ca/) (accessed on 23 July 2025) and the virulence factor database (VFDB) to predict genes related to antibiotic resistance and their associated virulence factor genes present in the genome. All homology searches were conducted under strict and conservative screening criteria to retain reliable alignment hits.

### 2.7. Statistical Analysis

All experiments were performed in in three independent biological replicates, and the data are expressed as the mean ± standard error (SE). ANOVA was employed to evaluate the significance of differences (with *p* < 0.05 considered to indicate statistical significance). Data visualization was performed using GraphPad Prism 10 software.

## 3. Results

### 3.1. Screening of Strains Producing Biosurfactants

**Figure 1:**
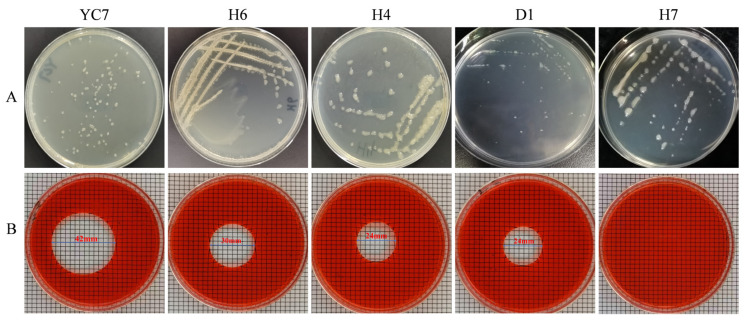
Screening results of biosurfactants-producing strains. (**A**) Colony morphology; (**B**) the results of oil spreading assay.

*Bacillus licheniformis* is the main microbial resource for producing lichenysin. Therefore, in this study, four strains of *Bacillus* were isolated from the core saccharifying and fermenting agent *Daqu* and the byproduct *Huangshui* during the brewing process of Baijiu. They were numbered as follows: D1 (from *Daqu*), H4, H6 and H7 (from *Huangshui*), and the colony morphology is shown in Figure 1A. The ability of the above strains (including the *Bacillus licheniformis* YC7 strain that has been preserved in our laboratory [22]) to produce biosurfactants was preliminarily determined by the oil spreading assay. The results showed that four strains had oil-displacement activity, among which two strains (YC7 and H6) have an oil spreading circle diameter greater than 30 mm (Figure 1B). The strain YC7 had the largest oil spreading circle diameter, reaching 42 mm, which was significantly larger than that of other strains, indicating that strain YC7 has a strong ability to produce biosurfactants. Therefore, YC7 was selected as the target strain for subsequent experiments.

### 3.2. Tandem Mass Spectrometry Analysis

**Figure 2:**
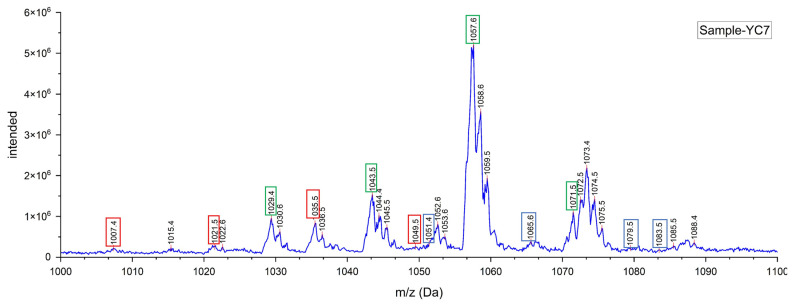
Primary mass spectrum of the liquid fermentation product of strain YC7. Red square labels indicate protonated molecular ion peaks of lichenysin homologues; blue square labels stand for sodium adduct ions [M+Na]^+^; green square labels denote disodium-substituted ions [M-H+2Na]^+^.

To identify what type of biosurfactants was produced by strain YC7 with strong oil displacement ability, the tandem mass spectrometry (MS/MS) analysis was performed on its liquid fermentation products. The full-scan mass spectrum of strain YC7 showed protonated peaks of putative lichenysin homologs at *m*/*z* 1007.4, 1021.5, 1035.5, and 1049.5 (Figure 2). Corresponding [M+Na]^+^ and [M-H+2Na]^+^ ions were observed at *m*/*z* 1029.4, 1043.5, 1057.6, 1071.5 and 1051.4, 1065.6, 1079.5, 1083.5, respectively. The ion peak with *m*/*z* value of 1015.4 is the corresponding [M+Na]^+^ peak of 993.4 [32,33]. These preliminary mass spectral signals suggested the possible existence of putative lichenysin analogues in the fermentation broth of strain YC7. Thus, MS/MS analysis was performed on ions at *m*/*z* 1007.4, 1021.5, 1035.5, and 1049.5 to characterize the structure of the main components.

**Figure 3:**
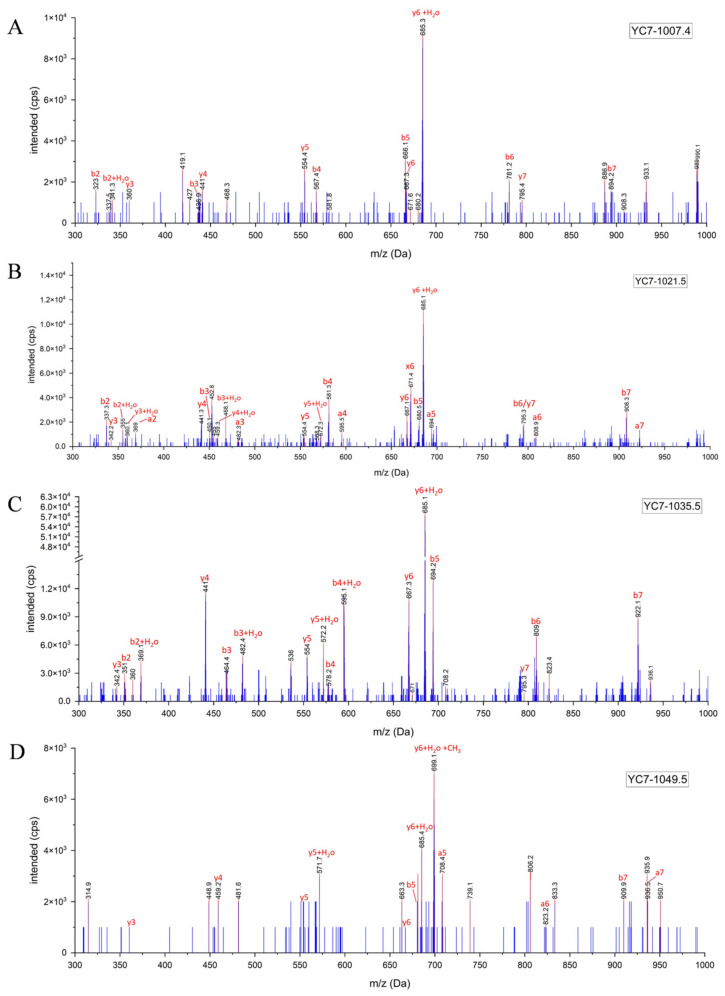
Secondary mass spectrometry of ion peak with *m*/*z* of 1 007.4 (**A**), 1021.5 (**B**), 1035.5 (**C**) and 1049.5 (**D**).

The MS/MS spectra displayed intensive b- and y-series ions for *m*/*z* 1007.4, 1021.5, and 1035.5 (Figure 3). Some fragment pairs differed by 18 Da, corresponding to dehydration during lipopeptide ring-opening, e.g., b2 at *m*/*z* 323 and 341.3, y6 at *m*/*z* 667.3 and 685.3. The most characteristic peak at *m*/*z* 685.3/685.1 represented the linearized peptide moiety after loss of the fatty acid chain, the first amino acid residue, and addition of one H_2_O molecule [34,35].

In Figure 3A, the ion at *m*/*z* 323 was assigned as [C13-OH-Gln+H^+^−H_2_O] [22]. Based on b2–b7 and y3–y7 ions, the lipopeptide was identified as lichenysin with a C13 fatty acid chain and the amino acid sequence, C13-Gln-Leu/Ile-Leu-Val-Asp-Leu-Leu/Ile, consistent with the structural characteristics of lichenysin A, B, or C subtype. Similarly, in Figure 3B, based on the ion peak characteristics of y3–7 and b2–7, as well as the prominent characteristic peaks at *m*/*z* 337.3 and 685.1, it can be inferred that it belongs to a homologue of lichenysin A, B, or C, with the structure C14-Gln-Leu/Ile-Leu-Val-Asp-Leu-Leu/Ile. In addition, there is a set of characteristic fragment ions a2–7 and x6 with *m*/*z* 671.1. The production of x6 was due to a double hydrogen transfer cleavage occurring at the Val at position 7, generating the characteristic fragment ion. Therefore, we tentatively inferred that this peak likely corresponds to putative lichenysin D/G homologues carrying a C15 fatty acid chain, with a proposed peptide sequence of C15-Gln-Leu-Leu-Val-Asp-Leu-Val. Figure 3C displayed a characteristic fragment ion at *m*/*z* 685.1. This fragment shared structural features with the ion at *m*/*z* 1021.4 and was assigned to a putative lichenysin homologue where the seventh amino acid was either Leu or Ile. We speculated that its tentative sequence is C15-Gln-Leu/Ile-Leu-Val-Asp-Leu-Leu/Ile, which can be categorized into putative lichenysin A/B/C subtypes. The MS/MS spectrum of the *m*/*z* 1049.5 ion is presented in Figure 3D. Its dominant fragment peak at *m*/*z* 699.1 matched a methylated y6 ion with one water adduct, which implies the C-terminal residue is most probably Leu or Ile, consistent with features of putative lichenysin A/B/C variants. From a5, a6 and a7 fragments, its candidate sequence can be putatively deduced as C15-Gln-Leu/Ile-Leu-Val-mAsp-Leu-Leu/Ile. Nevertheless, the limited coverage of a-type fragment ions reduces the confidence of this structural assignment.

### 3.3. Detection of Volatile Metabolites of Strain YC7

To explore the potential of strain YC7 to produce volatile substances through solid-state fermentation, we inoculated strain YC7 into wheat solid-state medium for fermentation and analyzed the types and contents of volatile substances in its fermentation products using GC-MS. Representative total ion chromatogram (TIC) of volatile metabolites produced by strain YC7 is presented in Figure S1. The types and yields of volatile compounds in the solid-state fermentation products of strain YC7 are shown in Table 1. The most abundant components were nitrogen-containing compounds, with a total content of 35.72 ng/g, including 2,5-dimethylpyrazine (16.72 ± 1.57 ng/g), 2,3,5,6-tetramethylpyrazine (10.67 ± 1.06 ng/g), and 2,3,5-trimethylpyrazine (8.33 ± 1.05 ng/g). Secondly, alcohols were present in relatively high amounts, with a total content of 19.3 ng/g. Among them, 2,3-butanediol had the highest content, reaching 11.13 ± 0.89 ng/g. In addition, ketones also had relatively high content, with a total of 17.17 ng/g. The ketone with the highest content was 3-hydroxy-2-butanone, also known as acetoin, with a content of 11.79 ± 0.95 ng/g. Acidic compounds also accounted for a certain proportion, with a total content of 16.29 ng/g, among which isobutyric acid was relatively abundant (15.05 ± 1.27 ng/g). Among phenolic aroma substances, guaiacol had a content of 5.33 ± 0.64 ng/g, and 4-tert-butylphenol had a content of 3.54 ± 0.46 ng/g. In the solid-state fermentation products of strain YC7, there were also various volatile aroma substances such as carboxylic acids, esters, and silanes.

### 3.4. Genomic Composition of Strain YC7

**Figure 4:**
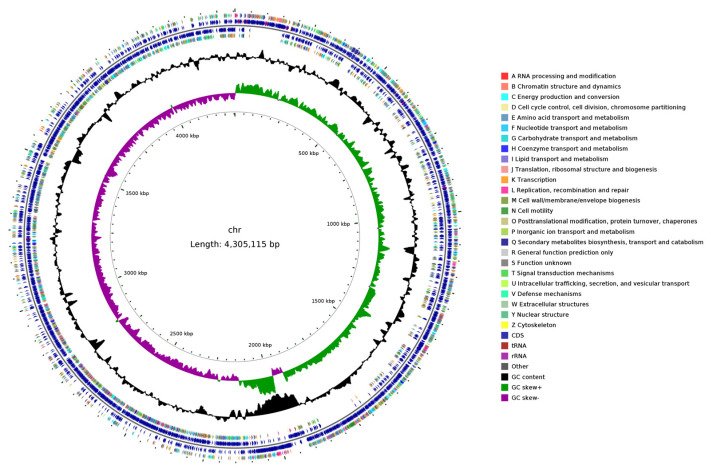
Genomic map of strain YC7.

To elucidate the molecular regulatory mechanisms underlying lichenysin biosynthesis and flavor metabolism in strain YC7, whole-genome sequencing was conducted. After sequencing and assembly, the complete genome sequence of strain YC7 was obtained (Figure 4). The full-length genome of strain YC7 was 4,305,115 bp, containing 4590 protein-coding genes, with a GC content of 45.93%. A total of 212 non-coding RNAs were identified, including 8 5S rRNAs, 8 16S rRNAs, 8 23S rRNAs, 83 tRNAs, and 105 ncRNAs.

### 3.5. Gene Function Annotation of Strain YC7

To reveal the metabolic characteristics, environmental adaptation mechanisms, and potential physiological functions of strain YC7, this study compared and annotated all predicted protein-coding gene sequences (CDS) against multiple public databases. The results showed that 6866, 4223, 4100, 172, 4564, 182, 36 and 54 genes were annotated in the GO, KEGG, COG, CAZy, NR, Rebase, VFDB and CARD databases, respectively.

#### 3.5.1. GO Annotation

**Figure 5:**
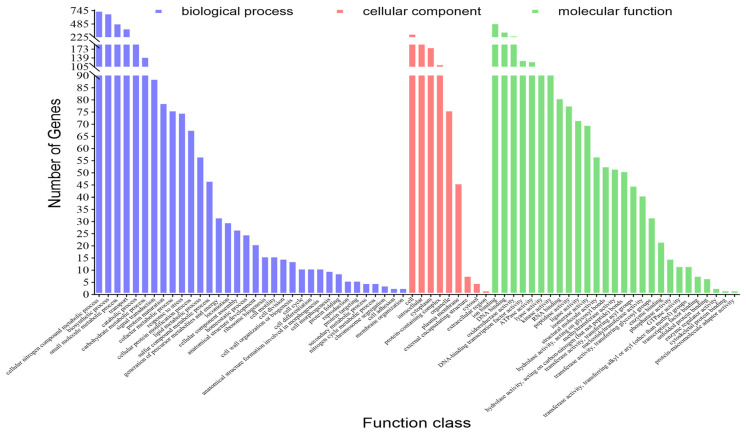
GO annotation of the genome of strain YC7.

GO annotation analyzes the relevant functional genes of strain YC7 from three dimensions: biological process, cellular component, and molecular function. The results showed that a total of 6866 genes of strain YC7 were annotated in the GO database, among which 3536, 2334, and 996 functional genes were annotated in biological process, molecular function, and cellular component, respectively (Figure 5). In the biological process, there were 43 genes associated with the synthesis of high-potential volatile flavor/secondary metabolites, including 18 core enzymes for volatile compound synthesis, 9 genes for transaminase activity, and 4 genes for branched-chain amino acid biosynthesis. Additionally, 34 genes were related to non-ribosomal peptide synthesis and modification, 92 genes to transport and secretion, and 155 genes to hydrolysis.

#### 3.5.2. KEGG, COG, and CAZy Database Annotations

**Figure 6:**
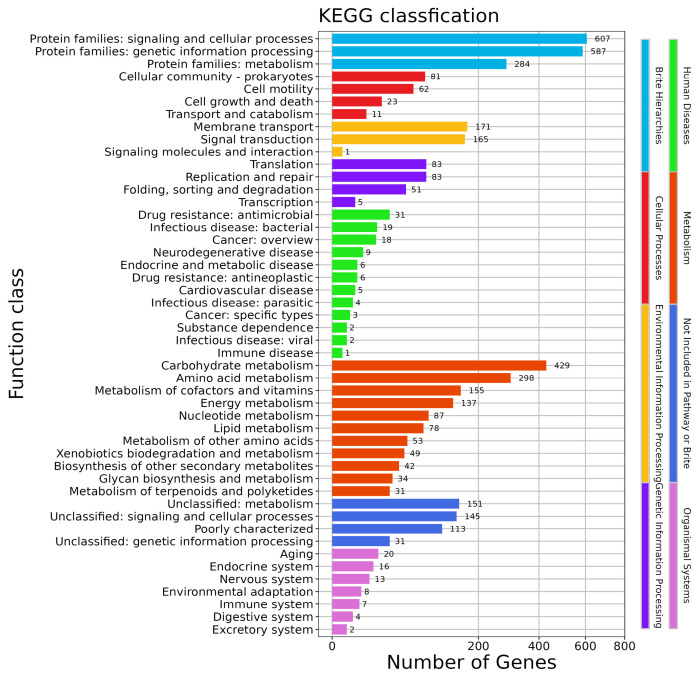
KEGG database annotation of the genome of strain YC7.

KEGG annotation was used to classify genes into functional pathways, systematically dissect metabolic pathways, signal transduction, and physiological functions, and construct a genome-wide metabolic network. The results showed that 1393 genes were involved in metabolism, 337 genes in environmental information processing, 222 genes in genetic information processing, 177 genes in cellular processes, 106 genes in human diseases, and 70 genes in organismal systems (Figure 6).

Focusing on lipopeptide biosurfactants (e.g., lichenysin), antibiotic biosynthesis, immunity, and drug resistance, the key pathways are listed in Table 2. Pathways *ko01053* (biosynthesis of siderophore group non-ribosomal peptides) and *ko01054* (non-ribosomal peptide structures) are responsible for the synthesis of lipopeptide biosurfactants such as lichenysin. Pathways including *ko00311* (penicillin and cephalosporin biosynthesis), *ko00332* (carbapenem biosynthesis), *ko00521* (streptomycin biosynthesis), and *ko00261* (monobactam biosynthesis) participate in antibiotic biosynthesis. Pathways *ko04621*, *ko04612*, *ko04659*, *ko04657*, and *ko05340* regulate immune and inflammatory responses. Drug-resistance-related pathways *ko01501*, *ko01502*, and *ko01503* correspond to beta-lactam resistance, vancomycin resistance, and cationic antimicrobial peptide (CAMP) resistance, respectively. Although these resistance pathways do not directly participate in antibiotic biosynthesis, they endow the strain with resistance to multiple antibiotics and represent key targets for safety evaluation.

**Figure 7:**
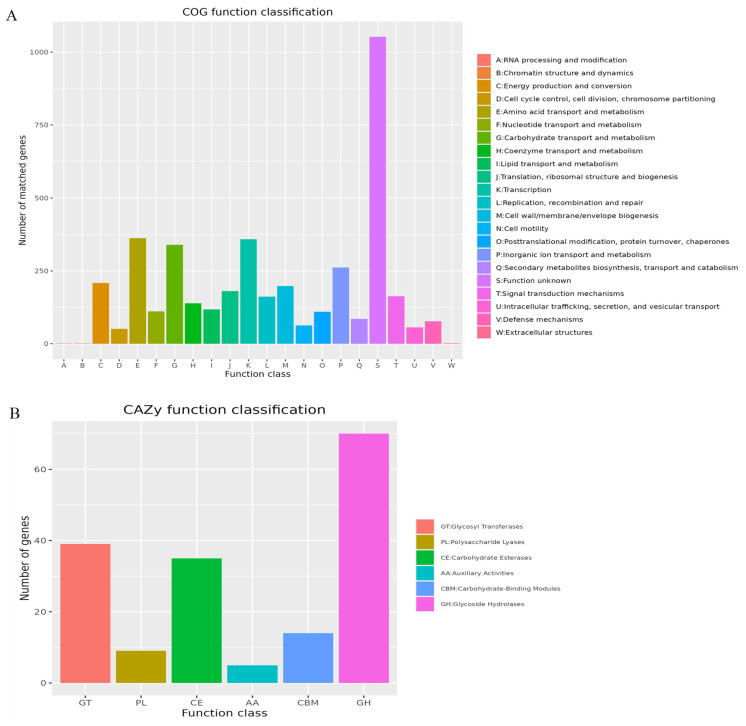
COG (**A**) and CAZy (**B**) database annotations of the genome of strain YC7.

To clarify the functional classification of protein-coding genes and reveal core features in metabolism and genetic information processing, COG annotation was performed on the genome of strain YC7. As shown in Figure 7A, 4100 protein-coding genes were classified into 22 COG categories, accounting for 89.3247% of the total protein sequences. Specifically, amino acid transport and metabolism contained 362 genes (accounting for 7.8867%); transcription contained 358 genes (7.7996%); carbohydrate transport and metabolism contained 339 genes (7.3856%); energy production and conversion contained 209 genes (4.5534%); translation, ribosomal structure, and biogenesis contained 181 genes (3.9434%); and lipid transport and metabolism contained 118 genes (2.5708%). In addition, genes with unknown function represented the largest proportion.

The CAZy database was used to annotate carbohydrate-active enzymes (CAZymes), including glycoside hydrolases (GHs), glycosyltransferases (GTs), polysaccharide lyases (PLs), carbohydrate esterases (CEs), and carbohydrate-binding modules (CBMs). As shown in Figure 7B, 172 CAZy-related genes were identified, covering six classes. GHs were the most abundant (70 genes, 1.53%), followed by GTs (39 genes, 0.85%), CEs (35 genes, 0.76%), CBMs (14 genes, 0.31%), PLs (9 genes, 0.20%), and auxiliary activities (AAs, 5 genes, 0.11%).

Based on family composition, gene abundance, and functional distribution (Table 3), the carbohydrate metabolism system of strain YC7 was comprehensive, target-specific, and coordinately regulated for synthesis and degradation. It showed functional bias in chitin metabolism, plant cell wall polysaccharide degradation, and endogenous polysaccharide biosynthesis. The significant variation in gene numbers and clustered distribution implied distinct functional importance and synergy among families, consistent with the enrichment of carbohydrate metabolism genes in COG annotation.

#### 3.5.3. Rebase Database Annotation

**Figure 8:**
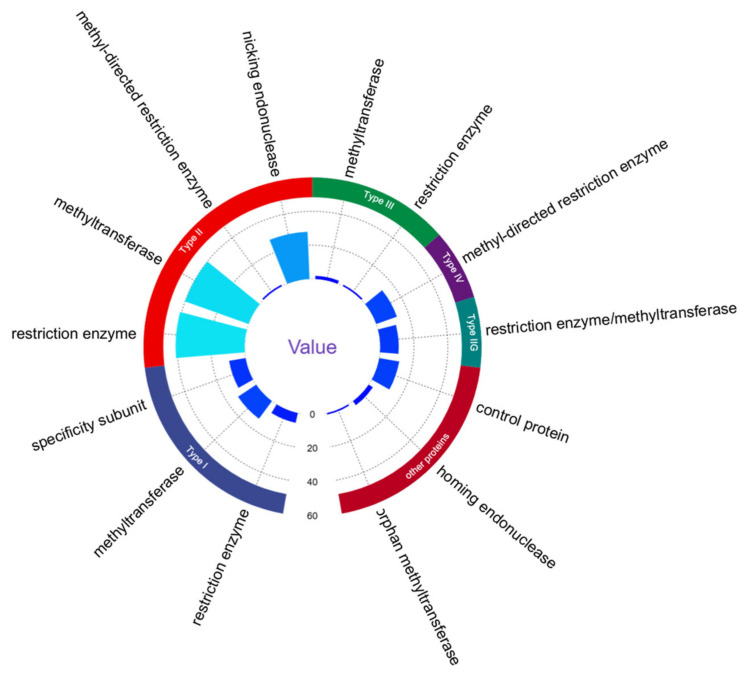
Rebase database annotation of strain YC7. This classification statistical diagram was manually drawn based on the Rebase annotation data output by bioinformatics analysis software.

The Rebase database was used to predict restriction-modification (R-M) system genes, including restriction endonucleases, methyltransferases, and complete R-M systems, revealing phage defense and epigenetic modification mechanisms for stress resistance and stability during fermentation. As shown in Figure 8, Type II-related enzymes (restriction, nicking, methyltransferases and methyl-directed restriction enzymes) numbered 110 in total, including 41 putative Type II restriction enzymes and 40 putative Type II methyltransferases, representing the major defense against foreign DNA. Type I-related enzymes numbered 29, requiring multi-subunit complexes to function. Type III/IV/IIG enzymes numbered 27, and 13 Type IV enzymes targeted modified DNA. The other 16 functional proteins included homing endonucleases, orphan methyltransferases and regulatory control proteins. These results indicated that strain YC7 possesses a complete R-M system dominated by Type II, which can effectively defend against foreign DNA invasion and improve environmental stability.

#### 3.5.4. Virulence Gene Prediction

Virulence gene annotation was performed to provide a molecular basis for safety evaluation of strain YC7. In total, 36 virulence genes were predicted, covering nine functional categories (Table 4). Specifically, immune modulation contained the most genes (13 genes, 36.11%), including *dep/capD*, *acpXL*, and *cpsA/uppS*, mainly involved in capsular polysaccharide synthesis and immune evasion. Nutritional/metabolic factors contained nine genes (25.00%), including *lplA1*, *pvdH*, and *dhb* family genes, related to siderophore synthesis, nutrient acquisition, and environmental adaptation. Stress survival contained four genes (11.11%), including *clpC*, *clpE*, and *clpP*, involved in proteasome-mediated stress response and protein homeostasis. Totally, four genes (11.11%) related to adherence, including *tuf*, *groEL*, *dnaK*, and *lap*, mediating adhesion and colonization. One gene each was found for exoenzyme (*eno*), exotoxin (*hlyIII*), invasion (*lpeA*), motility (*BCE_RS08700*), regulation (*sigA/rpoV*), and other functions (*icl*). Virulence-related genes of strain YC7 were mainly associated with environmental adaptation such as immune modulation, nutrition, adhesion, and stress response. No highly pathogenic virulence factors such as anthrax toxin or hemolysin were detected. Only one exotoxin homolog (hlyIII) was found, and very few exoenzyme or invasion genes existed. Most virulence homologs contributed to environmental adaptation, nutrient competition, and stress tolerance rather than pathogenicity. In summary, strain YC7 exhibited low virulence dominated by environmental adaptability. Considering the source of strain YC7 (from *Daqu*) and the long history of safe use of *B. licheniformis* in traditional fermented foods, it was inferred that strain YC7 has a high level of safety for food applications.

#### 3.5.5. Resistance Gene Prediction

The CARD database was used to predict and annotate antibiotic resistance genes, resistance mechanisms, and phenotypes, supporting safety assessment and stress resistance analysis. Strain YC7 carried 54 resistance genes involved in six resistance mechanisms (Table 5). Antibiotic target alteration was dominant (31 genes, 57.41%), conferring resistance by modifying or mutating targets such as ribosomes and DNA gyrase. Antibiotic efflux pumps contained 10 genes (18.52%), pumping antibiotics out of cells. Antibiotic inactivation contained eight genes (14.81%), encoding β-lactamases and aminoglycoside-modifying enzymes to degrade or modify antibiotics. Other mechanisms included target protection (one gene), target replacement (three genes), and reduced membrane permeability (one gene). Strain YC7 showed resistance to 23 types of antibiotics and disinfectants, including aminocoumarins, cephalosporins, diaminopyrimidines, etc. The multidrug-resistant characteristics of strain YC7 enable it to adapt to trace amounts of antibiotics or disinfectants that may be present in the fermentation environment, enhancing the stability of the strain during the fermentation process and ensuring the continuous synthesis of metabolites. Combined with the low-virulence characteristics identified through virulence gene analysis, this indicates that the strain’s drug resistance is only required for environmental adaptation, with no antibiotic resistance risk related to pathogenicity.

#### 3.5.6. Prediction of Secondary Metabolites

To predict secondary metabolite biosynthetic gene clusters, antiSMASH was used to analyze the whole genome sequence. Totally, 10 gene clusters were annotated in the genome of strain YC7 (Table 6). Three clusters showed 100% similarity to known clusters: Region 1 was the lichenysin non-ribosomal peptide synthetase (NRPS) cluster; Region 8 matched the bacillibactin (bacillibactin E/bacillibactin F) biosynthetic cluster; and Region 10 was a class-II lanthipeptide gene cluster.

#### 3.5.7. Prediction of Genes Related to Non-Ribosomal Peptide Biosynthesis

**Figure 9:**
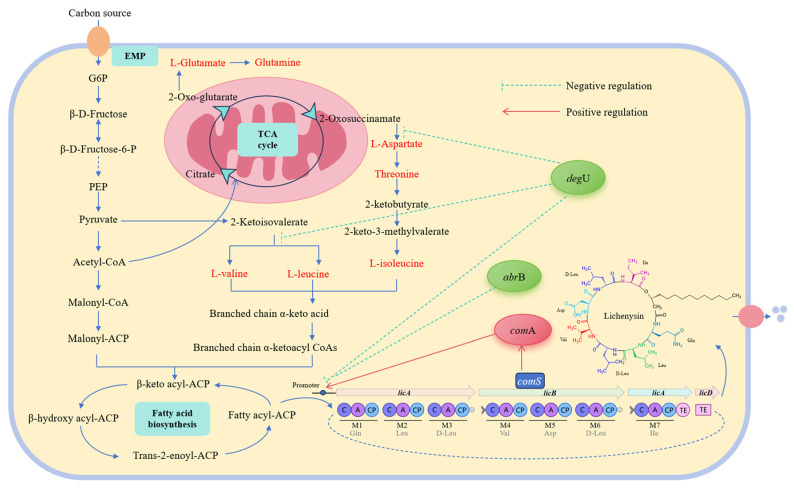
Overview diagram of lichenysin biosynthesis pathway. This schematic was manually plotted based on the whole-genome annotation data and metabolomic profiles of strain YC7 and published references on metabolic mechanisms [14,23,24].

Genes related to non-ribosomal peptide synthesis predicted by COG annotation are listed in Table 7. The annotated NRPS cluster includes key genes *licA*, *licB*, *licC*, and *licD* (homologous to *srfAA*, *srfAB*, *srfAC*, and *srfAD* in COG), which form the core framework for lichenysin biosynthesis and directly determine its structure and production capacity. In addition, quorum sensing and regulatory genes such as *comA*, *abrB* and *degU* can regulate the expression of lichenysin synthesis gene clusters through signal transduction. The lichenysin biosynthesis metabolic pathway is shown in Figure 9.

#### 3.5.8. Prediction of Genes Related to Volatile Metabolites Biosynthesis

**Figure 10:**
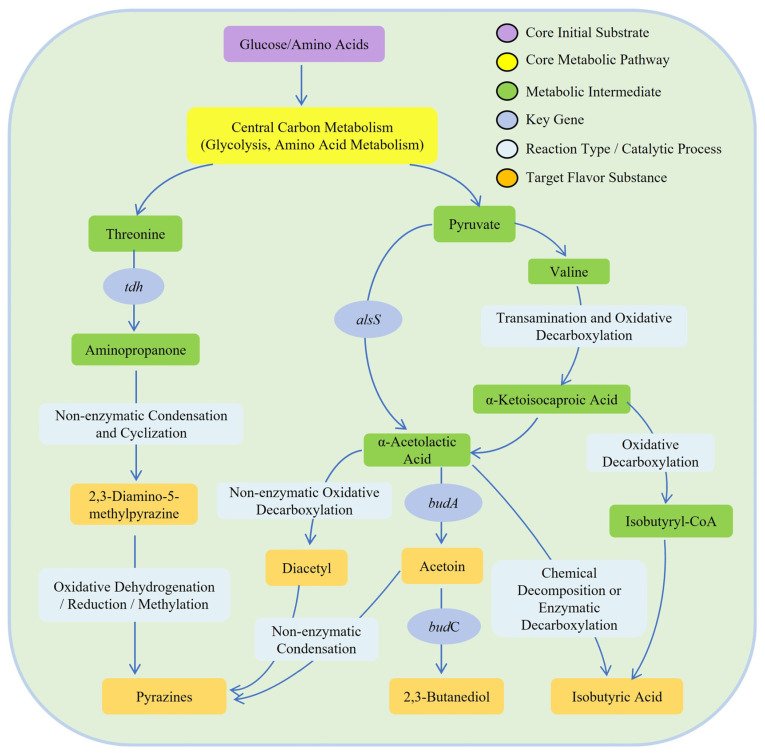
Biosynthetic pathways of the main volatile compounds in strain YC7. This schematic was manually drawn according to the whole-genome annotation data and metabolomic profiles of strain YC7 and published references [36,37,38].

COG annotation revealed that the biosynthesis of 2,3,5-trimethylpyrazine was associated with *tdh* (chr_1339, chr_1883) and *kbl* (chr_1884), while the biosynthesis of 2,5-dimethylpyrazine was additionally related to *pncB* (chr_3563) and *nadE* (chr_347) (Table 8). The *alsS* gene (chr_4067) can regulate the biosynthesis of acetoin and 2,3,5,6-tetramethylpyrazine. The 2,3-butanediol synthesis was controlled by *budA* (chr_4066) and *budC* (chr_2243) gene and isobutyric acid was regulated by the complete *ilv* gene cluster (*ilvA*, *ilvD*, *ilvC*, *ilvN*, *ilvB*, and *ilvE*). Inosine was regulated by the purine biosynthesis gene cluster (18 *pur* genes) and GMP metabolic regulatory genes (*guaB*, *guaA*, and *guaC*). The biosynthetic pathways of the main volatile compounds are shown in Figure 10.

## 4. Discussion

*Bacillus licheniformis* can synthesize lipopeptide substances such as lichenysin through the catalysis of non-ribosomal peptide synthetases. The oil-spreading assay showed that strain YC7 displayed better surfactant activity compared with the other tested strains (Figure 1). Tandem mass spectrometry further confirmed that it could produce lichenysin homologs A, B, C, and D with fatty acid chains ranging from C13 to C15 (Figure 3). The product profile highly matches the reported lichenysin profile of *B. licheniformis*, confirming that YC7 was a stable lichenysin-producing strain. However, the inability of MS/MS to differentiate isobaric leucine and isoleucine residues constrains definitive sequencing of lichenysin subtypes. Resolving these ambiguities will require future integration of high-resolution chromatography or amino acid analysis. Whole-genome analysis found that strain YC7 contained a complete lichenysin synthesis gene cluster. The antiSMASH prediction found that Region 1 was a 100% matching lichenysin NRPS biosynthetic gene cluster, containing key biosynthetic modules such as *licA*, *lic B*, *lic C* and *licD* (Table 6 and Table 7), which was the core genetic basis for its stable synthesis of lichenysin [22]. Meanwhile, COG annotations showed high-abundance genes related to amino acid and carbohydrate metabolism, as well as quorum sensing regulatory genes such as *comA*, *abrB*, and *degU*, jointly constructing a precursor supply and expression regulation network, ensuring efficient synthesis of lichenysin from multiple aspects, including material supply, regulatory pathways, and environmental adaptation [20].

The GC-MS results showed that strain YC7 can produce 35 volatile metabolites through solid-state fermentation. Nitrogen-containing compounds (35.72 ng/g), alcohols (19.3 ng/g), and ketones (17.17 ng/g) were the most abundant volatile flavor substances produced by YC7 (Table 1). Overall, the metabolic profile closely matches the flavor phenotype of functional *Bacillus* species in medium–high-temperature *Daqu* and the typical flavor characteristics of Baijiu [39]. Combined with whole-genome sequencing data, the abundant formation of these high-yield flavor compounds can be reasonably explained by the intrinsic functional genetic resources of YC7. Whole-genome analysis found that the synthesis of the advantageous products was supported by specific functional genes and metabolic pathways. The synthesis of pyrazine compounds relies on key genes such as *tdh*, *kbl*, *pncB*, and *nadE*. The TDH protein can catalyze the production of aminoacetone precursors from threonine. The function of KBL protein is to reduce precursor diversion, while pncB and nadE maintain metabolic balance by synthesizing NAD^+^, collectively enhancing the efficiency of pyrazine precursor supply [40,41,42]. The *alsS* gene can be involved in acetoin synthesis, and acetoin can react non-enzymatically with aminoacetone to produce tetramethylpyrazine [36,43]. The synthesis of acetoin and 2,3-butanediol is regulated by the *alsS-budA-budC* cascade pathway. The alsS catalyzes the conversion of pyruvate into α-acetolactate, which then leads to acetoin synthesis [44,45]. BudA enhances acetoin production, and BudC reduces acetoin to 2,3-butanediol [37,46]. This intact butanediol synthesis pathway accounts for the high abundance of alcohol substances detected by GC-MS. High production of isobutyric acid is closely related to the complete *ilv* gene cluster. The *ilv* gene cluster can regulate the efficient synthesis of the 2-ketoisovalerate precursor, and ilvE boosts isobutyric acid production through transamination [38]. Consistent with the high level of isobutyric acid in Table 1, the complete *ilv* operon may endow YC7 strong capacity to generate branched-chain short fatty acids. Additionally, the high-temperature, low-oxygen environment of solid-state fermentation in Baijiu can activate heat-resistant gene expression, speed up the non-enzymatic condensation of pyrazine compounds, and the starch and protein in the fermentation substrate provide plenty of carbon and nitrogen for product synthesis, further promoting the accumulation of key flavor substances.

Ten gene clusters encoding secondary metabolites were predicted (Table 6), including known clusters like lichenysin, bacillibactin, and class II thiopeptide lichenicidin, as well as three complete gene clusters with unknown function that might encode new terpenes, type III polyketides, or lasso peptides, suggesting that strain YC7 has the potential to synthesize new bioactive compounds. Studies have shown that lichenysin gives strains the ability to expand on hydrophobic interfaces [47], bacillibactin can inhibit the growth of competitors by chelating iron [48], and lichenicidin can produce antimicrobial peptides to strengthen the dominance of the microbial community [49,50]. These secondary metabolites might act as ‘chemical weapons’ and signaling molecules, working together to keep the strains active under high-temperature, low-oxygen fermentation conditions [47,48]. Additionally, strain YC7 contains 182 genes related to restriction endonucleases and methyltransferases, mostly Type II enzymes (Figure 8). This suggested that YC7 has a complete restriction-modification system, which can help it resist foreign DNA and enhance environmental adaptability [51,52].

By combining the genomic and phenotypic data of several previously reported *Bacillus licheniformis* strains, it was found that strain YC7 shows certain differences in metabolism. The model industrial strain ATCC 14580 is enriched with genes encoding industrial hydrolytic enzymes but lacks a complete pyrazine biosynthesis pathway and the ability to produce active surfactants [53]. The genomes of the livestock disease prevention strain JH41C and the wheat rhizosphere strain TRQ32 contain secondary antifungal gene clusters like fengycin and bacillomycin, but they lack flavor-related genetic elements and have no pyrazine accumulation phenotype [54,55]. The strain JXNUWL7 from fermented soybeans is only suitable for high-salt environments, has an incomplete pyrazine precursor synthesis pathway, and also lacks the regulatory genes for flavor compound synthesis [56]. The strain CGMCC 1.19452, isolated from sauce-flavored Daqu, can produce aroma at high temperatures but does not have strong emulsifying oil removal ability [57]. Compared with the above-mentioned strains, strain YC7 produced lichenysin and flavor substances and had a strong oil expulsion ability, which indicated that YC7 has a unique composite phenotype. Meanwhile, strain YC7 mainly carried virulence genes related to environmental adaptation, with no strong pathogenic factors. Its resistance genes were mainly mediated through target site changes, efflux pumps, and other mechanisms for environmental adaptation, matching the selection pressures from long-term low-dose disinfectants and fermentation products in the Baijiu brewing system. Therefore, it was speculated that this strain is likely to be quite safe and could potentially be used in the food industry.

Genomic annotation revealed the metabolic potential of strain YC7, yet these genome-scale predictions can only be correlated with partial metabolic phenotypes and fail to verify the in vivo activation and functional expression of related metabolic pathways. Further functional validation including transcriptomics, proteomics and targeted gene expression assays is still needed. In future research, multi-omics analysis can be adopted to dissect core metabolic regulatory mechanisms, laying a theoretical basis for the application of strain YC7 in fermentation and flavor modulation. The gene expression under different fermentation conditions could also be explored to identify core functional genes and key transcription factors that affect metabolic intensity. Additionally, fermentation scale-up tests could be gradually carried out to evaluate how well this strain adapts to real food industry fermentation scenarios.

## 5. Conclusions

The genome size of *Bacillus licheniformis* YC7 is 43,051,150 bp, with a GC content of 45.93%, and it encodes 4590 proteins. This strain can produce several lichenysin analogs under liquid fermentation conditions, and its genome carries a complete lichenysin biosynthesis gene cluster, indicating that YC7 has a stable ability to produce lichenysin at both phenotypic and genetic levels. Under solid-state fermentation conditions, YC7 can produce 35 kinds of volatile flavor compounds, with pyrazines being the dominant metabolic products. Additionally, it also produced high levels of acetoin, 2,3-butanediol, and isobutyric acid. Whole-genome analysis revealed that key functional genes like *tdh*, *kbl*, *pncB*, *nadE*, *alsS*, *budA*, *budC*, and the *ilv* gene cluster, as well as metabolic pathways like ko00650, ko00280, and ko00640, were annotated, showing a high coupling between the genome and metabolome. The genome of strain YC7 was rich in genes encoding CAZymes and also carries a lot of genes related to environmental adaptation. Its antibiotic resistance is mainly linked to environmental adaptation, the risk from virulence genes is low, and it has a complete Type II restriction-modification system. However, these functional interpretations were based on genome annotation and pathway prediction, which require additional experimental confirmation. Overall, these genomic features indicated that YC7 has the potential to meet the safety requirements for food-grade applications and stably colonize in complex fermentation environments.

## Figures and Tables

**Table 1:** Types and contents of volatile metabolites of strain YC7.

Classification	Flavor Compound	Content (ng/g)	Total (ng/g)
Nitrogen-containing compounds	2,5-Dimethylpyrazine 2,3,5-Trimethylpyrazine 2,3,5,6-Tetramethylpyrazine	16.72 ± 1.57 8.33 ± 1.05 10.67 ± 1.06	35.72
Alcohols	2,3-Dimethyl-4-penten-2-ol 2-Methyl-2,4-pentanediol 1,2,5,6-Dihydrouronic acid glycol Isooctanol 2,3-Butanediol 3-Methyl-2-heptanol Furfuryl alcohol	1.13 ± 0.22 0.92 ± 0.18 0.37 ± 0.09 2.06 ± 0.31 11.13 ± 0.89 0.32 ± 0.08 1.88 ± 0.28	19.3
Ketones	2-Nitro cyclohexanone 1,3-Dihydroxypropanone 3-Hydroxy-2-butanone	0.42 ± 0.09 4.96 ± 0.60 11.79 ± 0.95	17.17
Phenols	4-Tert-butylphenol Guaiacol	3.54 ± 0.46 5.33 ± 0.64	8.87
Acids	Isobutyric acid Trimethylpentanoic acid	15.05 ± 1.27 1.24 ± 0.21	16.29
Other flavor compounds	Methyl butyl nitrate Ethyl silicate	2.06 ± 0.35 1.45 ± 0.23	3.51
	Methyl silane	4.33 ± 0.65	4.33
	2-Nitro-2-chloropropane Methylhydrazine Inosine Benzaldehyde	2.26 ± 0.34 2.53 ± 0.38 0.23 ± 0.06 1.31 ± 0.22	6.33

Note: All content values represent the mean ± standard error (SE) of three independent biological replicates. The total value of each classification is the arithmetic sum of the average concentration of individual compounds within the group.

**Table 2:** Pathways related to the synthesis of lipopeptide biosurfactants, antibiotics, immunity, and drug resistance in strain YC7.

Pathway ID	Function	Gene ID
ko01053	Biosynthesis of siderophore group non-ribosomal peptides	chr_3447, 4118–4122
ko01054	Non-ribosomal peptide structures	chr_381–384
ko00311	Penicillin and cephalosporin biosynthesis	chr_258, 357
ko00332	Carbapenem biosynthesis	chr_1403, 1404, 2327, 2328
ko00521	Streptomycin biosynthesis	chr_955, 1646, 4472
ko00261	Monobactam biosynthesis	chr_443, 1741, 1861–1863, 2582, 3210, 4117
ko00401	Novobiocin biosynthesis	chr_2569, 2595, 2596
ko00405	Phenazine biosynthesis	chr_83, 171, 2602
ko00333	Prodigiosin biosynthesis	chr_1249, 1564, 1771, 1772, 1872, 3301
ko01051	Biosynthesis of ansamycins	chr_477, 478, 2212
ko00998	Biosynthesis of various antibiotics	chr_1773, 4163–4165, 4467
ko04621	NOD-like receptor signaling pathway	chr_2134, 3212, 3997, 4483
ko04612; ko04659; ko04657	Antigen processing and presentation; Th17 cell differentiation; IL-17 signaling pathway	chr_4483
ko05340	Primary immunodeficiency	chr_4232
ko01501	Beta-Lactam resistance	chr_173, 193, 255, 256, 258, 1058, 1214–1218, 2564
ko01502	Vancomycin resistance	chr_523, 524, 531, 1698, 1701, 2474, 3477
ko01503	Cationic antimicrobial peptide (CAMP) resistance	chr_160, 846, 1378, 1937, 2790, 3096, 3681, 4025, 4308–4311

**Table 3:** The core specific functional enzyme families of strain YC7.

Functional Types	Core Family (High Frequency *)	CAZy Type
Core for polysaccharide degradation	GH1 *, GH3, GH9, GH13 family *, GH43 family	GH
Chitin metabolism	GH18, GH23, CBM50 *	GH/CBM
Plant cell wall degradation	CE1 *, PL1 family *, AA1, AA6	CE/PL/AA
Core for polysaccharide synthesis	GT2 family *, GT4 *, GT8	GT
Polysaccharide de-modification	CE3, CE4, CE12, CE19	CE
Enzyme targeted localization	CBM9, CBM63, CBM92	CBM
Auxiliary oxidation for biomass degradation	AA1, AA4, AA6	AA

* Represents enzyme families with high expression frequency in strain YC7.

**Table 4:** Virulence genes of strain YC7.

VF Category	Number of Genes	VF Name
Immune modulation	13	*dep*/*capD*; *acpXL*; *cpsA*/*uppS*; *gndA*; *wcaJ*; *cps4I*; *galU*; *manA*; *capA*; *capC*; *capB*; *galE*; LPG_RS03745
Nutritional/Metabolic factor	9	*lplA1*; *pvdH*; *cbrD*; *mbtH*; *dhbF*; *dhbB*; *dhbE*; *dhbC*; *dhbA*
Stress survival	4	*clpC*; *clpE*; *clpP*; *clpP*
Adherence	4	*Tuf*; *groEL*; *dnaK*; *lap*
Exoenzyme	1	*eno*
Exotoxin	1	*hlyIII*
Invasion	1	*lpeA*
Motility	1	BCE_RS08700
Regulation	1	*sigA*/*rpoV*
Others	1	*icl*

**Table 5:** Classification of resistance mechanisms of strain YC7.

Resistance Mechanisms	Genes	Gene ID	Antibiotic Category
Antibiotic target alteration	*Saur pare AMU*; *Hpyl fxrA MTZ*	chr_2236; chr_787	Aminocoumarin antibiotic; Nitroimidazole antibiotic
	*Saur cls DAP*; *Bsub pgsA DAP*	chr_4114; chr_1876	Peptide antibiotic
	*Saur murA FOF, The tuL3 PLM*	chr_4177, chr_124	Phosphonic acid antibiotic; Pleuromutilin antibiotic
	*Ecol fabG TRC, Ecol fabI MULT*	chr_1772, chr_1249	Disinfecting agents and antiseptics; Disinfecting agents and antiseptics; Isoniazid-like antibiotic
	*Saur fusA FA*	chr_121	Fusidane antibiotic
Antibiotic target alteration/replacement	*Bsub rpoB RIF*	chr_116	Peptide antibiotic; Rifamycin antibiotic
Antibiotic efflux	*efrA*	chr_2349	Fluoroquinolone antibiotic; Macrolide antibiotic; Rifamycin antibiotic
	*ykkC*, *qacE*	chr_1398, chr_1921	Aminocoumarin antibiotic; Disinfecting agents and antiseptics
	*bacA*, *Efac iaR DAP*	chr_3499, chr_3692	Peptide antibiotic
	*lmrB*, *TaeA*	chr_2354, chr_732	Lincosamide antibiotic; Nucleoside antibiotic; Pleuromutilin antibiotic
Antibiotic inactivation	*aadK*, *mphK*, *catV*	chr_197, chr_275, chr_3042	Aminocoumarin antibiotic; Macrolide antibiotic; Phenicol antibiotic
	*BcIII*, *Saur GipT FOF*	chr_258, chr_4482	Cephalosporin; Penem; Phosphonic acid antibiotic
	*rphB*, *vgbC*	chr_2353, chr_237	Rifamycin antibiotic; Streptogramin B antibiotic; Streptogramin antibiotic
Antibiotic target replacement	*dfrG*	chr_2511	Diaminopyrimidine antibiotic
Reduced permeability to antibiotic	*IreK*	chr_1759	Cephalosporin

**Table 6:** Prediction of secondary metabolites of strain YC7.

Region	Cluster Type	From	To	Known Similar Gene Clusters	Similarity
Region 1	NRPS	363624	429073	Lichenysin	100%
Region 2	Thiopeptide, LAP	1024663	1065954	Butirosin A/Butirosin B	7%
Region 3	NI-siderophore	1178977	1212443	Schizokinen	60%
Region 4	Betalactone	2174726	2203240	Fengycin	53%
Region 5	Terpene	2299642	2321510	-	-
Region 6	T3PKS	2370845	2411942	-	-
Region 7	CDPS	3507016	3527765	Pulcherriminic acid	66%
Region 8	NRP-metallophore, NRPS	3803948	3855692	Bacillibactin/Bacillibactin E/Bacillibactin F	100%
Region 9	Lassopeptide, RRE-containing	3954010	3976743	-	-
Region 10	Lanthipeptide-class-ii	4040661	4067622	Lichenicidin VK21 A1/Lichenicidin VK21 A2	100%

**Table 7:** Functional genes related to non-ribosomal peptide synthesis in strain YC7.

Function	Gene	Gene ID
Non-ribosomal peptide synthesis	*srfAA*, *srfAB*, *srfAC*, *srfAD*, *sfp*, *dhbF*, *dhbB*, *entE*, *dhbC*, *dhbA*, *ubiE2*	chr_382, 383, 384, 387, 381, 4118, 4119, 4120, 4121, 4122, 298
Quorum sensing and regulation	*comA* *abrB* *degU* *degV* *comP*, *codY*, *degS*, *degT*, *degA*, *degR*, *degQ*, *spo0A*	chr_3555, 4258 chr_43, 1624 chr_422, 4010 chr_2505, 4009 chr_3556, 1797, 4011, 782, 1151, 2527, 3559, 2795

**Table 8:** Genes related to the synthesis of volatile compounds.

Compound	Gene	Gene ID
2,3,5-trimethylpyrazine	*tdh* *kbl*	chr_1339; chr_1883 chr_1884
2,5-dimethylpyrazine	*tdh* *pncB*; *nadE*	chr_1339; chr_1883 chr_3563; chr_347
2,3,5,6-tetramethylpyrazine	*alsS*	chr_4067
3-hydroxy-2-butanone (acetoin)	*alsS*	chr_4067
2,3-butanediol	*budA* *budC*	chr_4066 chr_2243
inosine	*guaB*; *guaA* *guaC*; *purR* *purE*; *purK* *purB*; *purC* *purS*; *purQ* *purl*; *purF* *purM*; *purN* *purH*; *purD* *purU*; *purA*	chr_10; chr_659 chr_1177; chr_53 chr_666; chr_667 chr_668; chr_669 chr_670; chr_671 chr_672; chr_673 chr_674; chr_675 chr_676; chr_677 chr_1400; chr_4550
isobutyric acid	*ilvA*; *ilvD* *ilvC*; *ilvN* *ilvB* *ilvE*	chr_2507; chr_2517 chr_3172; chr_3173 chr_3174 chr_3175; chr_4312

## Data Availability

The original contributions presented in this study are included in the article/Supplementary Materials. Further inquiries can be directed to the corresponding author.

## Supplementary Materials

The following supporting information can be downloaded at: https://www.mdpi.com/article/10.3390/foods15142548/s1, Figure S1: Representative GC-MS total ion chromatogram (TIC) of volatile metabolites of strain YC7.
