# Supplementary material for: Metabolites and Whole-Genome Analysis of the Lichenysin-Producing Bacillus licheniformis YC7
Source: Foods. 2026 Jul 19;15(14):2548. doi: 10.3390/foods15142548 (PMC13408588; doi:10.3390/foods15142548)
Supplement: Supplementary file 1 [file foods-15-02548-s001.zip › foods-4409492-supplementary.pptx]

## Slide 1
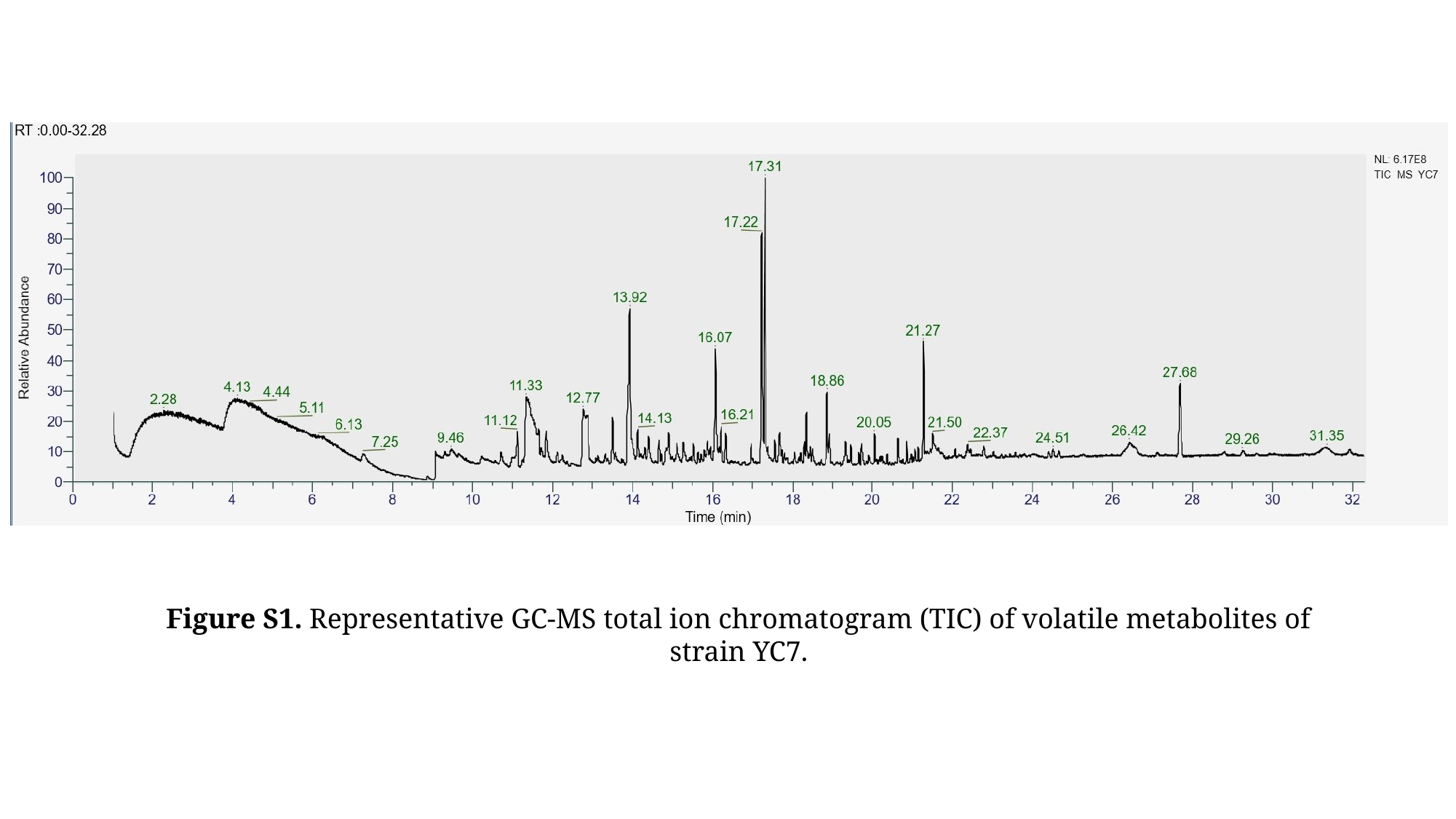

Figure S1. Representative GC-MS total ion chromatogram (TIC) of volatile metabolites of strain YC7.
